# Fail-tests of DNA methylation clocks, and development of a noise barometer for measuring epigenetic pressure of aging and disease

**DOI:** 10.18632/aging.205046

**Published:** 2023-09-12

**Authors:** Xiaoyue Mei, Joshua Blanchard, Connor Luellen, Michael J. Conboy, Irina M. Conboy

**Affiliations:** 1 Department of Bioengineering and QB3, University of California, Berkeley, Berkeley, CA 94720, USA; 2 Biophysics, University of California, Berkeley, Berkeley, CA 94720, USA

**Keywords:** DNA methylation, epigenetics, aging, clocks’ fail-tests, biological noise

## Abstract

This study shows that Elastic Net (EN) DNA methylation (DNAme) clocks have low accuracy of predictions for individuals of the same age and a low resolution between healthy and disease cohorts; caveats inherent in applying linear model to non-linear processes. We found that change in methylation of cytosines with age is, interestingly, not the determinant for their selection into the clocks. Moreover, an EN clock’s selected cytosines change when non-clock cytosines are removed from the training data; as expected from optimization in a machine learning (ML) context, but inconsistently with the identification of health markers in a biological context. To address these limitations, we moved from predictions to measurement of biological age, focusing on the cytosines that on average remain invariable in their methylation through lifespan, postulated to be homeostatically vital. We established that dysregulation of such cytosines, measured as the sums of standard deviations of their methylation values, quantifies biological noise, which in our hypothesis is a biomarker of aging and disease. We term this approach a “noise barometer” - the pressure of aging and disease on an organism. These noise-detecting cytosines are particularly important as sums of SD on the entire 450K DNAme array data yield a random pattern through chronology. Testing how many cytosines of the 450K arrays become noisier with age, we found that the paradigm of DNAme noise as a biomarker of aging and disease remarkably manifests in ~1/4 of the total. In that large set even the cytosines that have on average constant methylation through age show increased SDs and can be used as noise detectors of the barometer.

## INTRODUCTION

**Figure 1:**
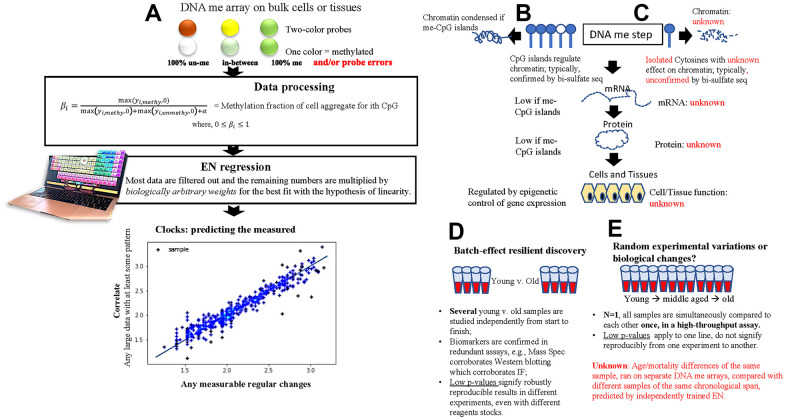
**Basic principles of the 1^st^ and next gen DNAme EN clocks.** (**A**) A schematic of the fundamental processing of DNAme arrays data into relative beta values and using these as input for the 1^st^ gen and next gen EN DNAme clocks. A large data set with some underlying pattern suffices for forming the least erroneous linear correlation with a measured parameter. (**B**, **C**) Comparison between the epigenetic analysis of gene expression in typical biological studies (**B**) and the cytosines picked by EN DNAme clocks (**C**). (**D**, **E**) comparison between typical biomedical studies that yield significant differences between cohorts in select parameters (**D**) and EN DNAme clock predictions (**E**). Of note, high throughput sequencing is broadly available, yet clock models are typically built from less-accurate DNA hybridization data, and parallel bisulfite sequencing controls are lacking for the putative changes in DNAme of the individuals who are predicted by the clocks to be biologically older or younger.

Elastic Net (EN) is the most used machine learning (ML) approach in constructing DNAme clocks [1–5]. The main principle of EN is to use regularization on datasets where the number of features far exceeds the number of samples and perform feature selection toward an absolute shrinkage of feature weights, allowing for the fit of a linear model to a relationship between the response variable and the predictors [6]. The EN model is trained using stochastic gradient descent to minimize a cost function (Equation 1) and returns a sparse set of feature weights, w^ (Equation 2). Prediction is given by taking a linear combination of the learned weights and a sample’s surrogate measure of methylation, e.g., the beta values (Equation 3) [7]. It must be noted that a least-error linear relationship is found regardless of whether the relationship is in fact linear. It is also notably different from typical biological experiments, that after most of the data are filtered out the remaining values are multiplied by weights that may be biologically arbitrary, as long as they mathematically enforce the hypothesis of linear change, Figure 1A.

DNAme clocks do not reflect the overall epigenetic state of a cell or a tissue and do not predict the process of epigenetic regulation of gene expression by CpG islands. Instead, the clocks are based on a small subset of cytosines that tend to be isolated and scattered sparsely through the genome, Figure 1B, 1C. Such disconnect of the clocks from the current understanding of gene regulation and purely correlative nature of predictions questions the relationship to the underlying biology. The DNAme array data are not derived from exact and well-controlled sequencing of methylated vs. unmethylated DNA. They are estimates of hybridization strength of 480-900K probes, read as optical signals of different intensity, Figure 1A. Probe hybridizations are largely based on the methylation status of a single end-of-probe cytosine. All signals are normalized and represented as beta values from 0 (unmethylated) to 1 (methylated), based on the maximum and minimum fluorescent intensities of a given cytosine read assay or Illumina run. Random differences in the hybridization efficiency of a probe and in the maximum and minimum fluorescent intensities of an assay or run are expected, and this influences the beta values and hence the predictions. After training on measured parameters, for example age or health scores, an EN model returns weights by which the beta values are multiplied before being summed; the sums are the correlate - predictor. There is no biological reason for weighting these beta values, but since the task is to correlate the measured axis linearly, EN returns the data-adjustment weights for a mathematical solution to the least-squares linear fit, Figure 1A [1–5].

The measured and then predicted axes are conceptually always a regular set of numbers (1, 2, 3… 10) be these inputted as chronological age (first generation clocks), levels of a few proteins followed by years-to-death (GrimAge), a composite of clinical parameters associated with age (PhenoAge), a health scale for people in ~26-50 age range (PACE), or any measurable parameter. In EN, no physical meaning is implied to these numbers.

As expected, EN regression can confirm well-known, robust differences of age, disease, ectopic epigenetic determinants such as Yamanaka factors, trisomy, etc., cases where DNAme arrays and bisulfate sequencing yield similar data [8–12]. However, artifacts of non-specific probe hybridizations are expected to increase with age and disease due to DNA damage and mutations (C to T and others), skewing the data distribution in the outputs of DNAme arrays [13, 14]. Aging and disease also involve lymphoid to myeloid, fibrotic, inflammatory, senescent, and other shifts; these may be accurately determined by Flow cytometry, single cell RNA Seq, ATAC-Seq, and *in situ* omics, but are unresolvable in bulk DNAme arrays [15–17].

In typical biomedical research, a statistically robust number of samples is studied independently from start to finish, however, the metrics of clock performance (e.g., mean squared error, median absolute error, p-value) are often correlations based on an experimental n=1, Figure 1D, 1E. Low p-values mean that the best-fit line could not have been generated by chance in this one experiment, but do not signify accuracy from one experiment (dataset) to another. The natural dynamics of DNAme and experimental differences in instruments, reagents stocks, researcher introduced, etc. [18–21] are inherent and largely uncontrolled for, meaning that after a successful DNAme array assay, no input/output of EN is known to be more or less accurate than another, and none normalizes for the other. The dataset (split into a training and test subsets) is a subject to above-mentioned batch effects, and thus, the possibility of misinterpreting random experimental variation for biologically relevant changes. The reliance of clocks solely on hybridization arrays also contrasts with typical molecular biology approaches that study each parameter through several redundant assays [22].

The idea of a biological age clock has been explored over the past decades through studies of the Hayflick number, telomere length, DNAme and proteomics, all under the hypotheses that each of these methods reflects a life-long linear change of a biomedically important parameter. Clock’s cytosines have been profiled as biomarkers but such bioinformatics seems to be currently de-emphasized [3, 23]. With respect to the notion of linearity, many studies demonstrate a healthy young-adult plateau with stably controlled epigenetics, gene expression and protein levels [24–27]. And not all patterns of aging are determinants, for example, hair greying, or loss might be age and health-linked and can be used in EN-based predictions, but hair restoration does not yield health or youth and hair is not a determinant of mortality [28, 29].

Diseases contribute to health decline and shorten life span; the diseases of age can be detected by many well controlled analyses, including genetics, epigenetics, and clinical tests without clocks that, in some cases, differ from clock predictions [30–33]. The question is not whether epigenetics can be used to detect changes in health or if health declines with age, compounded by diseases (both are given), but whether ML clocks predict these changes with high resolution or as early as other methods.

Here, we reveal the capacities and limitations of DNAme clocks of all generations, by expanding the n=1 experimental to a more systematic examination of clock performance on many different DNAme array datasets of healthy controls and patients with various diseases. Additionally, we show that even though EN regression models linearly predict age and risk of mortality from DNAme array data, the clocks are not based on any specific changes in DNA methylation with age, or dysregulation of such methylation, e.g., a change in SD of beta values. Lastly, we describe a conceptually different measurement (not a prediction) of persons’ biological age, which we term a noise barometer, reflecting the concept of *pressure* of aging and disease.

## RESULTS

### The robustness of linear correlations varies over the age ranges

We found that while EN-trained DNAme models yield high linear correlation (r > 0.9) between age and DNAme over the complete age range, weaker linear correlations manifest in tests on binned age ranges (Supplementary Figure 1), from 0.16 ≤ r ≤ 0.8. Specifically, bins of 18-30yr (during the maturation of the immune system) and 80-100yr have the highest correlation of r=0.8, with the rest of the age ranges are more moderately correlated (0.16 ≤ r ≤ 0.5). The uneven correlations of different age bins to DNAme changes are consistent with the notion of non-linear changes in biological age over the lifespan, e.g., the healthy plateaus and steeper health decline after combinatorial tissue disrepair [24–27].

A non-linearity of epigenetic changes through the lifespan should be expected, due to genetic and environmental variability between people, compounded by the high dynamics of epigenetics that is influenced by such parameters as sense of well-being, financial status, circadian rhythm, etc. [34, 35].

The non-linearity of biological age, the high variation of the assay and, hence, of the data, which is used for EN training, might result in diminished accuracy of correlations, particularly for some age groups. On the other hand, the overall estimates of being young, middle-aged, or old are reliably well predicted by the EN DNAme clock.

### Tests of accuracy and biological relevance of predictions

Two claims are made by studies of EN-based predictors, first that the errors in prediction by DNAme clocks are attributed to age acceleration in particular individuals. In support for this attribution, a similar acceleration was seen for some diseases, albeit only for some age groups [1–5, 36]. A second claim is that because clocks trained on one sample set can work on out-of-sample set data, this indicates some biological relevance of EN predictions and of cytosine selection.

**Figure 2:**
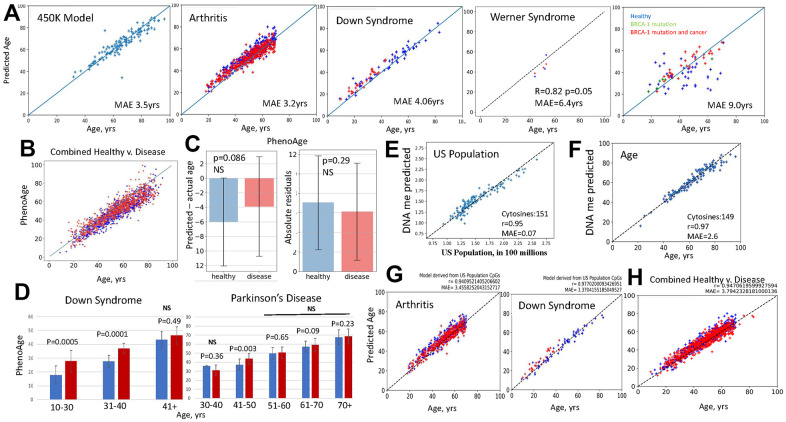
**Fail-tests of EN models that are trained on biological v. non-biological parameters.** (**A**) Standard first generation EN model was trained on 450K DNAme arrays dataset; tests with the original and new datasets, as indicated, are shown (blue – healthy, red – disease). BRCA-1 studies were done on 27K dataset, thus, after training on 27K dataset. EN was tested on BRCA-1 data. EN models were testable with the original and new datasets, except for the BRCA-1, where only the subjects with BRCA1 mutation and cancer but not healthy controls received fairly accurate age predictions. All EN models overlapped predictions for healthy subjects and patients (see Supplementary Figure 2 for the tests of EN models on additional disease v. healthy datasets). (**B**) PhenoAge scatter plots overlay for the patients with the studied datasets (red dots-Combined Disease, blue dots-Combined Healthy). (**C**) PhenoAge tests on the six 450K datasets are represented as the bar graphs on Mean and SDs of predicted age minus actual age (left) and as the comparison in absolute residuals (right); patients – red bars n=806, healthy controls – blue bars; n=754, p-values are shown, NS- non-significant. (**D**) PhenoAge predictions of DNAme age for the indicated age-intervals are shown as bar graphs of Means with SDs for the patients with Down Syndrome and Parkinson’s Disease (red bars), and their healthy controls (blue bars). DS, n=26, Control, n=58; PD, n=289, Control, n=219; p-values are shown. (**E**) EN model was trained on the US population numbers, at subjects’ birth years, using the same 450K DNAme array, as in A. The test shows excellent linearity and low MAE in predicting the numbers of people living in US from DNAme array, through 151 clock cytosines. (**F**) The US population EN model was then trained to predict persons’ age with successful tests (near perfect linearity and MAE of 2.6 years) by 149 clock cytosines. (**G**) This US population clock was also testable as an age predictor on the out-of-sample 450K datasets of indicated diseases (red dots) and their healthy controls (blue dots); health and disease predictions overlapped. (**H**) The age predictions made by the US population clock are shown as scatter plots overlay for Combined Disease (arthritis, Down Syndrome, HIV and Chron’s – red dots) and their healthy controls (Combined Healthy, blue dots). All GSEs are in Methods.

To examine these claims, we performed systematic fail-tests of DNAme clocks with large DNAme datasets from nine independent studies. First, we reconstructed EN regression models that replicate the published first-generation clocks, and tested them with the original and out-of-sample DNAme array datasets, in which healthy subjects were compared to those with disease: Rheumatoid arthritis, Down syndrome, BRCA-1 cancer, Werner syndrome, Multiple Sclerosis, Crohn’s Disease, Ulcerative Colitis, Irritable Bowel Disease, and an HIV dataset that did not have a parallel healthy control, Figure 2A and Supplementary Figure 2. Model performance was assessed by correlation coefficient (Pearson’s r) between the predicted and actual ages and median absolute error (MAE) (Equations 4, 5).

The models fairly accurately predicted the age of samples, but with an error of age acceleration throughout chronology (shifted above the line) and with overlap in age predictions for the patients and healthy subjects, Figure 2A and Supplementary Figure 2. This is biologically inconsistent, as arthritis significantly increases PBMC inflammaging, [37, 38], Werner Syndrome is a disease of premature aging (caused by a mutation of DNA helicase) [39, 40], and Down syndrome has a pathological juvenile blood phenotype with a prevalence of childhood leukemias and less mature circulating PBMCs [41]. In tests on BRCA-1 studies, the EN model had very high MAE and poor correlation with the age of the healthy subjects but produced fairly accurate predictions for the people with the BRCA-1 mutation, and cancer, Figure 2A.

The 27K EN model that was from the combination of disease and healthy samples surprisingly reported age acceleration when tested on the 450K dataset that was from relatively healthy individuals, Supplementary Figure 2. 27K dataset cytosine probes are present in the 450K dataset. Lastly, we found that a simple ordinary least squares regression (OLS) on the set of clock’s cytosines also yielded linear correlations, and these were somewhat more accurate in predicting age (lesser age acceleration through chronology) than the EN model, Supplementary Figure 2.

In testing PhenoAge, thought to be biologically more relevant, we expanded the approach to multiple independent studies with 450K DNAme arrays on Arthritis, Multiple Sclerosis, Down Syndrome, Parkinson’s, IBS, Werner Syndrome. We analyzed whether predicted age is on average higher for disease samples as compared to their healthy controls. Additionally, instead of MAE on one 450K correlation, which is the Median from an experimental n=1, we quantified the errors as the arithmetic Means of predicted minus actual age, with Standard Deviation (SD) and p-values, in these six large data studies, experimental n=6. In other words, we applied the typical data analysis that is used in biomedical research, when testing for a relevant change.

When applied to the DNAme clock, these analyses demonstrated that PhenoAge generally predicted that both healthy subjects and patients with various diseases were younger than their chronological age (shifted below the line) and these predictions had on average 12-17 years of error, which for some age groups grew to over 20 years, Figure 2B, 2C. The accuracy of PhenoAge was below statistical significance when it was analyzed with n=6, based on the errors for the same age and when comparing health v. disease. This agrees with batch effects causing a lack of reproducibility, and/or with the normally high variation in the underlying biology of epigenetics. PhenoAge predicted Down Syndrome patients to be biologically older than controls, even though their blood cells are reported to be pathologically juvenile [41]. Parkinson’s disease patients surprisingly had a prediction trend of being younger than their healthy controls at 30–40-year range and not statistically different from their healthy controls at all age intervals after 51years, Figure 2D.

The second-generation clocks are trained on, and can thus successfully predict, various biological and health parameters and also age, in both test and out-of-sample datasets. As mentioned above, this property of EN does not in itself require a biological meaning or even a physical connection between the correlated and the measured parameter. To demonstrate this point, we constructed a EN clock that accurately predicted the numbers of people living in US from DNAme array data, and like PhenoAge and PACE, it also predicted people’s age on both the original and out-of-sample datasets, Figure 2E–2G. In fact, the US population clock needed only 149 cytosines for predicting age with 2.6-year MAE and Pearson’s r of 0.97, e.g., outperforming many conventional DNAme clocks, Figure 2E. The DNAme predictor of the size of US population also illustrated that the ML EN process tends to overlap age predictions for health and disease, regardless of the training variable, Figure 2G, 2H. The increase in US population over time is a proxy for chronology, but this is also true for all DNAme clocks, be they trained on age, levels of inflammatory cytokines, packs of cigarettes smoked, composite health scores, or any metric which tends to have a progressive change with time. The meaning of the values that are composed together to create a latent variable are not preserved in EN training. EN regression only requires that there are some features which have at least some moderate correlations with time progression.

**Figure 3:**
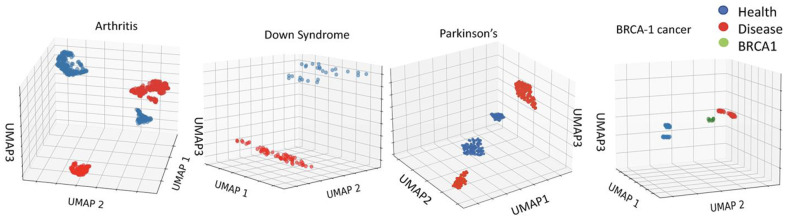
**Robust epigenetic differences between health and disease are clear in the 450K datasets.** Distinct clustering of DNAme of healthy vs. disease cohorts. UMAP on the 450Kcytosinedataset for Arthritis, Down Syndrome, Parkinson’s Disease and BRCA1 studies (see Methods for dataset identifiers). Each point represents an individual. The clustering of healthy, cancer, and BRCA1 mutation yet no cancer cohorts is distinct for each dataset.

As expected from the body of published literature, inflammaging, trisomy and diseases are robustly different from health, and this can be detected with much higher resolution than EN by analyzing the primary, un-adjusted, non-linearized data of the DNAme array. For example, Uniform Manifold Approximation and Projection (UMAP), which preserves the topological structure of the data (with all 450k+ cytosines) while embedding it in multi-dimensional computational space, robustly distinguishes between health and disease in the studied here 450K array datasets, Figure 3 [42].

Thus, the input DNAme data accurately reflects the expected significant epigenetic differences between healthy and disease populations. However, under the training of a least erroneous linear correlation EN is expected to minimize the weights of cytosines-beta values, which vary among individuals of the same age (or its proxy) due to a disease. Even when a combination of health parameters is used for training, as with the next generation EN clocks, the disease-imposed DNAme differences that vary between age groups, might be overlooked in search for the best linear predictors of age or age-related outcomes for the whole population.

Summarily, if chronology or its proxy is the response variable, and finding the best linear correlation is the task, the relevance of ML predictors is not obvious. Additionally, random experimental variation of the arrays and/or EN training might be misinterpreted as biologically meaningful change in all generations of clock models.

### EN predictors are not based on change in DNA methylation with age

**Figure 4:**
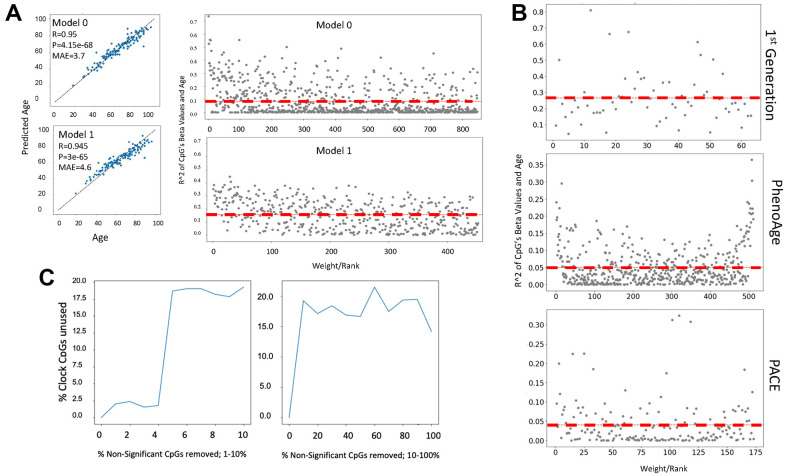
**Cytosine ranking by EN is not based on the changes in methylation with age.** (**A**) DNAme clock was constructed with EN regression on a 450K array dataset (GSE40279, N=656), and the test set prediction performance is shown (Model 0). The selected clock cytosines were removed, the model was retrained, and model performance on the test set is shown (Model 1). Scatter plot of the coefficient of variation for each clock cytosine individually regressed on age plotted against their rank by their absolute weights. The mean coefficient of variation of the clock cytosines are shown as red dashed lines. The cytosines of Model 1, e.g., those which were minimized to zero in Model 0, have a higher mean R^2^, despite being slightly less accurate. (**B**) Scatter plots of the coefficient of variation for the cytosines regressed on age are plotted against their rank/EN weights of the published 1^st^ generation Hannum clock, PhenoAge and PACE. (**C**) An EN clock was trained with a 450K dataset (GSE40279), then non-clock cytosines were randomly removed: independently in a stepwise fashion. After each iteration of removal, a new EN model was trained and the selected set of cytosines was compared to the set of original clock cytosines. Non-clock cytosines were removed in one percent increments from 0-10% (left panel) and 10% increments from 10-100% (right panel). At 2% of removal of the non-clock cytosines, the cytosine set selected by EN began significantly changing. The percentage of unused original cytosines plateau at 17% in both gradual (1% at a time) and rapid (10% at a time) removal of the non-clock cytosines.

To explore a possible mechanism by which clock features predict age or health scores we studied the assigned weights of cytosine beta values of several published DNAme clock models. The weight assigned by a model to a predictor is a surrogate measure of its importance in the model, as the predictions returned by a model are most sensitive to changes in predictors with the highest weights. Interestingly, we found that EN does not necessarily select cytosines whose methylation either change or correlate strongly with age, and hence are biologically meaningful in such a way, Figure 4A.

In fact, the ranking of cytosines as per their methylation change with age became *better*, yet MAE became slightly worse, after all the original clock picked cytosines were removed from the dataset, and as expected, EN selected the next weighted best fit linear predictor set, Figure 4A. The redundancy of cytosine sets is known, e.g., EN is “resilient” to specific cytosines, or their combination, and it is the EN process on large data that gives the linear correlation. In support of these finding, the changes of DNAme with age were not the determinant of the ranking of cytosines by the published 1^st^ generation clock trained on age or PhenoAge, and PACE trained on health/disease parameters [1–5], (Figure 4B). The red dashed lines are the mean coefficient of variation and that the line remains flat with age signifies the irrelevance of age-specific changes in DNAme for the ranking of clock’s cytosines.

Exploring the mechanisms of cytosines selection further, we next removed not the clock picked cytosines, but the cytosines that were excluded from the model by EN, and then retrained EN on the remaining set, and we did this repeatedly, Figure 4B. Removal of unpicked cytosines significantly changed the picked cytosines from the original training, plateauing at 17-20% of the unused original set after removal of 3-4% of irrelevant cytosines. DeepmAge, PhenoAge and Hannum’s 71 clock cytosines were similarly deselected upon the non-clock cytosine removal, Supplementary Figure 3A–3C.

Such behavior does not agree with a hypothesis that biomarkers should not change when non-biomarkers are deleted from a dataset and supports the null hypothesis that picked cytosines are not biomarkers. ML methods that rely on stochastic gradient descent for optimization appear to suffer from the pitfall that removal or absence of certain features changes the hyper surface of the feature space which influences the local optima (i.e., feature sets, weights and hyperparameter values) that the gradient descent can converge upon. This point was experimentally supported by studying the effects of removing the non-significant cytosines on the energy landscape of EN, Supplementary Figure 3D.

Summarily, the biomedical significance of clock cytosines is not clear, and DNAme clocks are not directly based on the changes in DNA methylation with age or disease.

### It is not absolute beta values, but the noise (SD) of certain cytosines that biomarks biological age

In the final part of our study, we quantify biological age from the DNAme arrays data, using six independent studies with 450K DNAme arrays that were performed on 1806 samples. We postulated that homeostatically vital cytosines would serve well as the detectors of biological noise, which in our hypothesis is a biomarker of aging and disease. Furthermore, we postulated that such noise detecting cytosines can be characterized and thus identified by a nearly equivalent Mean of their beta values between young and old, but a higher SD about the Mean with age. Our goal was to measure this indicator of biological age, not to predict it.

**Figure 5:**
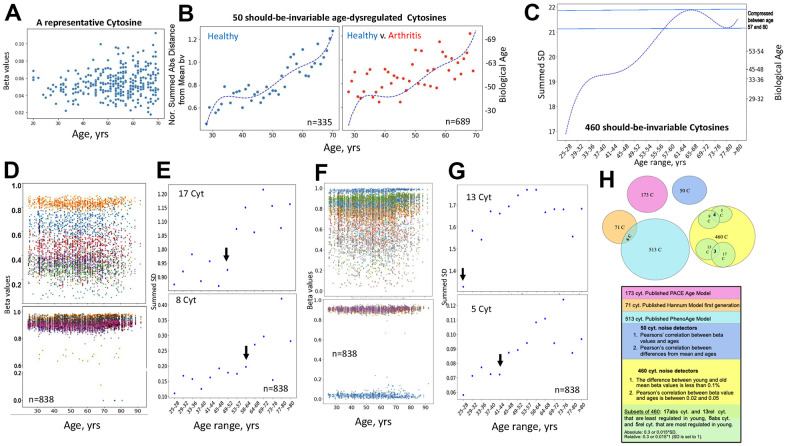
**Direct quantification of biological age from DNAme array data through noise barometer. **(**A**) Representative cytosine that is on average invariable in its methylation throughout lifespan but becomes visibly noisier, showing higher absolute deviation from the mean in older individuals. Each dot is an individual. (**B**) Polynomial curve was fitted to the dot-plot of Median of normalized by healthy young sums of SDs of the 50 cytosines for healthy individuals, left; the same polynomial curve is overlayed with the dot plot of the Median of normalized by healthy young sums of SDs of the 50 cytosines for arthritis patients, right. Biological ages were mapped onto the right Y axis, as described in Methods. Each dot is age range: blue – healthy, red, arthritis. (**C**) Polynomial curve was fitted to the 460 Summed SDs (of the 460 cytosines) v. chronological age ranges, using the six combined 450K DNAme datasets of healthy controls. Right Y axis shows mapping of the summed SDs into biological ages and the compression of specific age ranges. (**D**) Scatter plots on the changes in beta values over age for representative cytosines that are least regulated in young, with SD=0.3 of absolute Mean of beta values, and all 8 most regulated in young cytosines with SD=0.015 of absolute Mean of beta values. Each dot is a sample. (**E**) Dot plots of the summed SDs of the 17 cytosines and of the 8 cytosines. Each dot is 49 samples on average. Black arrows indicate transitions from low to high noise. (**F**) Same as (**D**), but for cytosines that have SD=0.3 and 0.015 of the *relative *Mean of beta values. (**G**) Same as (**E**), but for the summed cytosines with SD=0.3 and 0.015 of the *relative* Mean of beta values. (**H**) Venn diagrams of the cytosines of the published clocks and the 460, 50 and 5, 8 – most regulated, 13, 17 least regulated cytosines of the noise barometer that quantifies biological age; the text is color-coded per each cytosine set.

The age-imposed increase in the DNAme noise is visible when looking at a scatter plot of the beta values of individual cytosines that are on average invariable in their methylation across the lifespan, as exemplified in Figure 5A (each dot is an individual). To capture this biological phenomenon from the DNAme array data, we first specifically identified the cytosines that remain on average invariable in their methylation through age (Pearson’s (r) 0.02-0.05 of beta values over age) and become deregulated or “noisy” with age (change in absolute distance of their beta values from the Mean, ADM); this yielded 50 cytosines out of the 450K DNAme array, GSE42861 dataset, Figure 5B. Consistent with the hypothesis of biological relevance, all these cytosines turned out to be in vital genes known to be homeostatically regulated, Table 1 and Supplementary Data Excel, which shows genome loci annotations of the 50 noise detector cytosines. The biological age (Right Y axis) was then quantified based on the Median of the normalized by the young age sums of SDs of these 50 cytosines (Left Y axis). This unadjusted primary data had a good fit with polynomial curve, it displayed age-imposed increase in biological noise with a plateau at ~35-45 years of age, in healthy subjects, and a clear increase in the noise for the young and middle-aged patients with arthritis, Figure 5B (each dot represents the indicated age; the raw data with dots representing individuals per each age is shown in Supplementary Figure 4A).

**Table 1:** Families and functions of the genes annotated to the 50 cytosines noise biomarkers.

**Probe**	**Gene**	**Family**	**Effects**
cg00868523	PRKCG	Protein Kinase C	Pleiotropic
cg01767885	C1QTNF8	C1q/TNF-Related	Cell motility - vital
cg01869186	EPHB4	Ephrin receptor/RTK	Vascular repair - vital
cg01900413	ETS-1	TF	Pleiotropic
cg02247160	CTNNB1	Beta-catenin	Pleiotropic
cg04153991	PHLDB2	Regulators of cadherin	Cell adhesion and cytoskeleton - vital
cg04495670	FGF3	FGF, MAPK signaling	Pleiotropic
cg06362282	B3GALT4	Membrane-bound Galactosyltransferase	Pleiotropic
cg06494091	GNAQ	Guanine nucleotide-binding protein	Pleiotropic
cg06918474	3-Mar	Membrane associated ring-CH-type finger	E3 ligase - Pleiotropic
cg08499046	Pax6	TF	Pleiotropic
cg12615535	RBP1	Carrier protein	Retinol transporter - Pleiotropic
cg12717533	FAM59B	GRB2 Associated	Regulator of MAPK1 - Pleiotropic
cg12779520	CD6	T-Cell Differentiation Antigen	Continuation of T cell activation
cg13399816	GNG12	G protein-coupled receptor signaling	Pleiotropic
cg13966628	FMN2	Formin homology	Actin cytoskeleton, cell polarity - Pleiotropic
cg14993491	PCSK9	Proprotein Convertase	Regulate the amount of cholesterol in the bloodstream - vital
cg15556672	MRPS36	Ribosomal proteins	Mitochondrial Ribosomal Protein S36 - vital
cg17096412	TMEM98	Transmembrane proteins	Secreted form promotes differentiation of Th1 cells Negative regulator of MYRF
cg17216583	RPL22	Ribosomal proteins	Ribosomal protein component of the 60S subunit - vital
cg19355182	PDE4A	Phosphodiesterase	hydrolyzes cAMP - Pleiotropic
cg20261915	GLP2R	G protein-coupled receptor	Glucagon receptor - vital
cg20283716	ASPSCR1	Tether with UBX Domain	GLUT4 regulation / insulin response - vital
cg23417875	MAP4K4	MAPK signaling	Activates JNK - Pleiotropic
cg23801965	GSC2	Goosecoid-like homeodomain	Axis formation - Pleiotropic
cg23983449	GABARAP	GABA R associated proteins	Regulates ligand-gated chloride channels and neurotransmitter signaling
cg24027320	TASP1	Endopeptidase	Maintenance of HOX and TFIIA gene expression Pleiotropic, vital
cg24112454	KIAA1409	Ionic channels	accessory subunit of the NALCN channel that contributes to the Ca2 sensitivity - vital
cg27621745	CHMP4C	Chromatin modifying /charged multivesicular body family	MVB formation and regulation of cell cycle progression - Pleiotropic
cg27642618	CD33	Sialic-acid-binding Ig-like lectin	Cell-cell interactions Pleiotropic. Resting state of immune cells.
cg07165260	KIAA0513	Uncharacterized	Uncharacterized
cg16443148	CCDC78	Coiled-coiled domain	Unknown
cg25007283	ZIC4	C2H2-type zinc finger	Not well characterized
cg00479463	*	*	*
cg01681847	*	*	*
cg05207943	*	*	*
cg06899313	*	*	*
cg07710266	*	*	*
cg12518535	*	*	*
cg14400541	*	*	*
cg14657277	*	*	*
cg20037507	*	*	*
cg20445245	*	*	*
cg24201362	*	*	*
cg25734842	*	*	*
cg27219748	*	*	*
cg27317439	*	*	*

* Not characterized.

Next, we examined if DNAme noise, which we define as the age-imposed increase in SD of beta values, would self-manifest when we select cytosines solely based on their nearly-same methylation through chronology. We identified all the cytosines with less than 0.1% difference in the Means of their beta values between the young (25-28years) and the old (67+ years) individuals, in the healthy controls’ datasets of the studies on Down Syndrome, Arthritis, Parkinson’s, Multiple Sclerosis, IBS, and Werner Syndrome. A healthy change in cytosine noise is biologically expected as the immune system matures [43, 44], thus our young range is 25-28 years. These cytosines that are well-regulated through age were also cross-checked by having Pearson’s (r) 0.02-0.05 of beta values over age, e.g., excluding the zeroes of experimental errors; this approach yielded 460 cytosines that had minimal change in their Mean methylation with age. Interestingly and in agreement with our hypothesis, every one of these cytosines, whose methylation on average was age-invariable, manifested an increase in SD – DNAme noise with age, Figure 5C and Supplementary Data Excel. This unadjusted primary data had a good fit with a polynomial curve of chronological ages v. summed SD (Left Y Axis); and the non-linearity of the pattern compressed certain biological ages, which were mapped onto the Right Y axis by entering the summed SDs into the polynomial equation, Figure 5C.

Next, we narrowed down the 460-cytosine set of the noise detectors to those which are most regulated in the young cohort and those least regulated in the young cohort; once again, identifying the cytosines with these properties that are shared between all studied six healthy controls’ datasets. Out of 450K array data, only 460 cytosines had nearly the same Mean of beta values in young and old cohorts, and out of these only 8 had SD of >0.015 of the *absolute* Mean and only 5 had SD of >0.015 of a *relative* (set to 1) Mean of their beta values, e.g., they were the most regulated. Very interestingly, all the 8 and 4 out of the 5 were highly methylated (beta values ~1) and the remaining one cytosine had beta value near 0 – unmethylated; none were in-between or partially methylated, even though the samples analyzed for cytosine methylation were of a mixed population of peripheral blood cells, Figure 5D, 5F.

It is possible that these cytosines reflect loci that should be always completely silenced (say, controlling a different cell fate) or open (say, for housekeeping genes), regardless of the cell types of the circulating cells. In contrast, the cytosines that were the least regulated in the young cohort, 17 cytosines with SD of <0.3 of the *absolute* Mean and 13 cytosines with SD of <0.3 of a *relative*=1 Mean, had a large range of methylation (spanning the 0-1 range of beta values), Figure 5D, 5F. Other least regulated cytosines are shown in Supplementary Figure 4B. Annotations for the 5, 8, 13 and 17 cytosines – noise detectors are in Supplementary Data Excel.

Summing the 8, or the 5, or the 17, or the 13 Means of the SDs for each 3-year age interval between 25 and over 80 years, established that each set detected a natural pattern of human biological aging with a progression of epigenetic noise that is low in young, high in old, and has healthy, low noise plateaus, Figure 5E, 5G (each dot is 49 individuals on average, black arrows point to the 3-year interval before the upward transitions in noise). Interestingly, both the least and most regulated cytosines, which were identified as such by the two approaches in the young samples, became noisier with age, Figure 5E, 5G. 64-67 years and 49-52 years demarcated the upward shifts in biological noise (black arrows) for the 8 most- and 17 least-regulated cytosines, respectively, (the SD of the *absolute* Mean of beta values), Figure 5E. 41-44 years and the very early 29-32 years transitions from low to high biological noise (black arrows) were detected for the 5 most- and 13 least-regulated cytosines, respectively (SD of the *relative* Mean of beta values), Figure 5G.

We think of this measurement of noise increase with age as a barometer, implying epigenetic pressure on homeostatic gene regulation.

The 460 cytosines that had constant Means with age and the 50 cytosines found on average invariant yet noisier with age by linear regressions, were independent of the first-gen, PhenoAge and PACE EN cytosine sets, Figure 5H. The young-to-adult focused PACE cytosine set was also expectedly different from the full age-range focused first generation and PhenoAge models.

**Figure 6:**
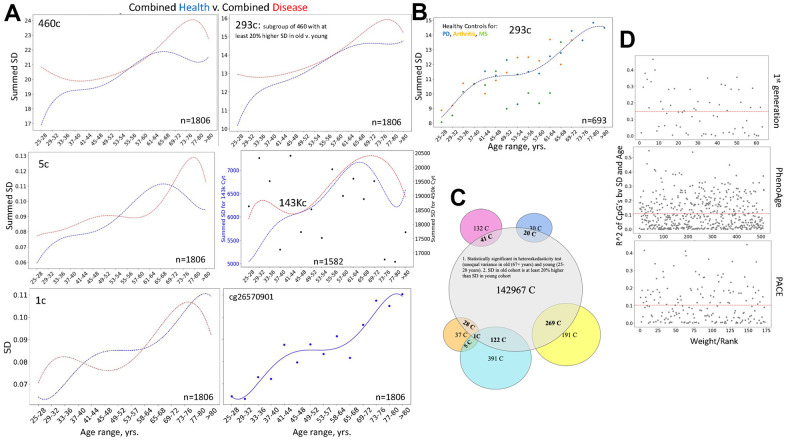
**The fundamental paradigm of noise barometer; DNAme clocks do not rely on DNAme noise. **(**A**) Shown are polynomial fit curves of the chronological age v. sums of 460-, 293-, 5 SDs, SD and the dot plot line-fit of a single cytosine from the most regulated in young group, and the sums of 143, 448 SDs of all heteroscedastic cytosines with 20% or larger SD in old than young; the unfiltered 450K summed SDs are the black dot scatter that is overlaid with the 143, 448 (143K) line graph. These data are on the six combined datasets of patients with various diseases (red lines) and their healthy controls (blue lines). All noise detectors, but not the unfiltered 450K DNAme cytosines, outline a similar progression of biological aging of the healthy subjects, clearly distinguishing it from the disease-influenced changes. Each dot is the age range. (**B**) Dashed blue line shows the Median of summed SDs of three DNAme array healthy controls datasets that have the most samples (Arthritis, MS, and PD); this Median line is overlayed with the dot plots of each of these individual datasets, color-coded. Each dot is the age range. (**C**) Venn Diagrams show the presence of the 143K cytosines in the 50 and 460 noise barometers and in the DNAme EN clocks, color-coding is the same, as in Figure 5. (**D**) Scatter plots of the coefficient of variation of the cytosines’ SD regressed on age are plotted against their rank/EN weights for the published 1st generation clock, PhenoAge and PACE, [1–5].

Next, we examined differences between health and disease, using the combined DNAme array data from the six studies by different laboratories. As mentioned, all 460 cytosines of noise barometer self-manifested higher SD of beta values in the old as compared to young, and 293 of these cytosines had at least 20% increase in their SDs with age. Interestingly and consistently with the fundamental paradigm of DNAme noise as a biomarker of aging and disease, summing the SDs of 460, 293 or 5 cytosines yielded similar patterns of healthy biological aging and the disease-imposed shifts, Figure 6A. Moreover, this pattern of biological aging and difference between health and disease were also yielded by SD of a single noise barometer cytosine (one of the most regulated in young), Figure 6A. The overlap between health and disease at certain ages is expected from the overall elevation of diseases with age (not just those studied), and from the possibility of *pathologically diminished* biological noise in some disease stages due to reduced complexity of blood cells (less lymphocytes and greater prevalence of inflammatory macrophages).

Confirming that selection of noise detecting cytosines is important, sums of SDs of all cytosines from these six combined 450K DNAme array healthy controls’ datasets showed a random scatter through chronological age, Figure 6A, each black dot is an age range. At the same time, in agreement with the fundamental nature of the noise / SD approach, 1/4^th^ of 450K DNAme array cytosines (143,448 cytosines) was found to be significantly heteroscedastic and having 20% or more increase of the SDs of their beta values with age. Summing the 143,448 SDs yielded a similar pattern of biological aging and disease-imposed shifts, as when using 460, 293, 5, or 1 cytosine(s), Figure 6A.

Similar shapes of the healthy curves and good resolution between health and disease in the combined data from six independent studies by different laboratories suggested batch effect resilience of our noise barometer. This was confirmed and extrapolated by comparing the three most numerous healthy control datasets (from the studies of Arthritis, Parkinson’s, and Multiple Sclerosis) in their biological aging curves, Figure 6B and Supplementary Data Excel. Low batch effect was seen for the three individual healthy datasets, except in the 49-64 years age range, which could be due to a natural increase in DNAme dynamics from cumulatively diverging environments, habits, and diseases (not just those specifically studied), Figure 6B. Based on Venn Diagrams, 460 cytosine noise detectors have the highest overlap with the global 143 thousand subset, followed by the 50 cytosine noise detectors and various EN DNAme clocks, Figure 6C and Supplementary Data Excel.

The pattern of healthy biological aging based on the 460 and 5 cytosines was also similar to that yielded by the 50 cytosines, Figures 5B, 6A. And the 50 cytosines noise barometer demonstrated different curve shapes for different diseases, Supplementary Figure 5A. At the single levels, the most regulated cytosines – noise detectors had different patterns of their age-specific noise increase, Supplementary Figure 5B and Figure 6A. The natural biological aging curves of the combined health vs. combined disease that include the HIV dataset, are shown in Supplementary Figure 5C.

Since SD is the main parameter in our noise barometer, we also tested if EN clocks might be based on selection of cytosines that have higher SD of their beta values with age and/or disease. The regression analysis demonstrated that this is not the case for either the 1^st^ generation EN clock that is trained on age or for PhenoAge or for PACE that are trained on health/disease parameters [1–5]. Namely, there was a lack of correlation between the ranking (weighting) of the clock’s cytosines by the ENs and the changes of the SDs of their beta values with age, Figure 6D.

These results suggest that biological age can be measured in a batch effect resilient approach through quantification of the SD of the methylation of cytosines that should-be-invariable, yet are age deregulated. The natural curve of biological aging defined by this approach is not linear; it has healthy low-noise plateaus and upward shifts at specific ages. In this method, DNAme noise is the biomarker and a particular set of cytosines is the detector of such biomarker. This method is different from and moreover, orthogonal to, the linear predictors that are based on population statistics. Importantly, regardless of the selection of all the cytosines that should-be-invariable (50, 460) or their subsets most or least regulated in young (1, 5, 8, 13, 17), an increase of biological noise with age was always observed, and there was never a high in young to low in old progression for any cytosine of the noise barometer.

## DISCUSSION

In this work, we fail-tested the key concepts of ML, EN DNAme clocks, and as such, our conclusions should be broadly applicable to most/all variants of these approaches. Our data confirms that the general phenomenon of change with age, e.g., the distinction between young, middle age and the old, is accurately determined by ML population statistics. With respect to overall implications and unmet need, changes in health are routinely quantified by Student’s t-test, etc. statistics, of well-controlled, independently analyzed from start to finish, epigenetic, proteomic and other molecular and cellular analyses of blood samples. These allow single cell resolution and are confirmed through redundancy of experimental methods, guarding against misinterpretation of random variability or typical dynamics for the changes in health.

Standard biomedical analyses giving Mean, SD, and p-values from several independent start-to-finish studies do not permit the erroneous assumption that a sample from each person is an experiment in itself when a population was studied by aligning all samples, once. Through such standard analyses, we illustrate how batch effects and dynamics of DNAme might reduce the reproducibility and accuracy of the clocks, making these, at times, below statistical significance. Of note, small, poorly reproducible changes might appear significant, and the healthy group cannot be reliably used as a standard for a disease group, when p-values are calculated to diminish from the sheer number of samples in an n=1 study.

Regarding fundamental conclusions, it is well known that a 75-year-old person with high levels of C-reactive protein, or diminished albumin, or with obesity, or high cholesterol, is unhealthy and has an increased risk of mortality. EN regression finds correlation, not causality, and as we show here, it does not rely on the changes of cytosine methylation or dysregulation with age or disease. Thus, even though DNA me array data is used, the biological meaning of predictions remains uncertain. And as we illustrate with the size of US population, non-biological changes can be successfully predicted by the DNAme array datasets. Summarily, any set of regularly changed numerical values can be linearly predicted through EN training on any large data, biological or not, physically connected to the measured axis or not. If anybody is interested, an expansion of the Universe could be predicted from evolutionary DNAme array data. And a person’s health and age could be predicted with relatively good accuracy from expanding distance between the Galaxies, or anything that changes progressively over time.

Since the physical meaning of the training parameters is lost in EN process, any assumption that specific health scores, C-reactive protein levels, etc., are being predicted, is ambiguous, because in the model training, a decline in health, reaching mortality and the increase in age all have linear progressions designated as a regular series of numbers.

In these regards, high throughput sequencing is routine, yet clock models are typically built from less-accurate DNA hybridization data. For reliable scientific or consumer information from EN and other ML approaches, several key controls are important, such as, 1) performing independent start to finish experiments with the same or near-same-time samples, that are analyzed on independently run DNAme arrays, and compared with different samples of the same chronological span, by independently trained EN models; 2) sequencing-normalized bisulfite sequencing controls on whether a clock’s cytosines change in their methylation when a person is predicted to be age/disease accelerated or at 2-4 years age intervals; 3) confirming the chromatin change near clock cytosines by ATAC-Seq, for example; 4) testing whether genes of clock cytosines change their expression (mRNA and protein levels) when a shift in biological age is predicted for a person; 5) confirming that the levels of specific proteins (GrimAge) or health scale are statistically different in 4-2-years ranges, or at the point of age acceleration.

Through our investigation, we describe a new quantification of biological age from DNAme, based on the noise of the most-regulated, age invariable cytosines. This approach fits well with the importance of age-specific increase in biological noise [45] and it is the quantification of primary data without numerical adjustments, which improves on ML predictions. The points of increased DNAme noise turned out to be 49-52 and 64-67 years of age, and it would be very interesting to probe global omics at these transitional ages. Our noise barometer distinguishes health from disease and can potentially distinguish one pathology from another completely different pathology. The different time-shapes of different diseases might enable epidemiology of a specific disease in a population, based on the curve of epigenetic noise. The biological significance of noise-detector cytosines is clear and the effects of their deregulation with time and disease are expected to be many and deleterious. Namely, the likely reason for the noise-detectors cytosines to be on average invariable in their methylation with age is that they are in the regulatory regions of genes that are vital at constant levels, such as coding for ribosomal proteins, core transcriptional factors, E3 ligases, regulators of cholesterol and glucose metabolism, MAPK signaling, beta-catenin, Table 1. The less-characterized loci of noise detector cytosines, Table 1 and Supplementary Data Excel, would be interesting to study for their involvement in biological aging. The paradigm shift is that no specific cytosine or gene is biomarks biological age, but it is the noise and dysregulation, e.g., it is not the expression levels or beta values, but the SD that are the basis of quantifying biological age and risk of disease. Accordingly, summing SD of either 143 thousand or 460, or 5 cytosines. or even SD of one cytosine noise detector, yields overall the same non-linear curve of DNAme aging and distinguishes health from disease. The noticeable plateaus of the DNAme aging emphasize that biological age is progressing non-linearly, in contrast to the chronological age, making accurate linear predictions problematic for certain age ranges. Quantification of the deviation from the healthy range seems to be more relevant and it is enabled by the noise barometer.

Reasonably accurate predictability of chronological age from DNA samples positions EN models as potential forensics tools, to inform on the age-ranges of suspects, as was indeed suggested. DNA methylation arrays enable rapid high throughput analysis and generally agree with bisulfate sequencing when the big changes are assayed, such as differences between cancer vs. cancer-free cohorts [46–48]. Yet, for a law enforcement degree of accuracy, it might be prudent to also perform higher resolution bisulfite sequencing, controlled by non-bisulfite sequencing, for testing the precision of the age predictions.

Lastly, the assignments of human biological age are typically based on blood samples, and while some patterns might be shared in different organs (for example, fibrosis and inflammation generally increase with age, which is not healthy) the overall biological age is determined by the *integrated* dynamics of divergent cell-fate gene regulation in the organism, which varies between the tissues, collectively influencing organismal metabolism, proteostasis, organelles, fibrosis, regeneration, etc.

The above should not be confused with predicting ages of different tissues and species [49]; in a similar fashion, changes in human health, population, and temperature can be collectively predicted, or any set of regularly changed numbers, through training EN on DNAme array or any large data.

## MATERIALS AND METHODS

### Methods

Code protocols are at https://github.com/jeblanchard/DNAme-clocks-fail-tests-and-development-of-noise-barometer?search=1

### Datasets

Small (27K) model datasets

GSE41037: WB, Schizophrenia and health patients, https://www.ncbi.nlm.nih.gov/geo/query/acc.cgi?acc=GSE41037

GSE20067: WB, type 1 diabetes with nephropathy and type 1 diabetes without neproprathy,

https://www.ncbi.nlm.nih.gov/geo/query/acc.cgi?acc=GSE20067

GSE20236: WB, healthy females,

https://www.ncbi.nlm.nih.gov/geo/query/acc.cgi?acc=GSE20236

GSE19711: WB, postmenopausal women with ovarian cancer and healthy controls,

https://www.ncbi.nlm.nih.gov/geo/query/acc.cgi?acc=GSE19711

Medium (450K) model datasets

GSE40279: WB, no labeled diseased samples - can interpret this to be an average population,

https://www.ncbi.nlm.nih.gov/geo/query/acc.cgi?acc=GSE40279

Out-of-sample (450K) datasets

GSE57285: WB, BRCA1 set, cancer, healthy mutants, and healthy wild types,

https://www.ncbi.nlm.nih.gov/geo/query/acc.cgi?acc=GSE57285

GSE53840: WB, HIV set, all HIV+ males,

https://www.ncbi.nlm.nih.gov/geo/query/acc.cgi?acc=GSE53840

GSE42861: PBL, Rheumatoid arthritis set, rheumatoid arthritis and healthy controls,

https://www.ncbi.nlm.nih.gov/geo/query/acc.cgi?acc=GSE42861

GSE32148: Peripheral blood; Crohns’ disease, ulcerative colitis, and normal controls;

https://www.ncbi.nlm.nih.gov/geo/query/acc.cgi?acc=GSE32148

GSE52588: WB, Down syndrome and healthy relatives as controls,

https://www.ncbi.nlm.nih.gov/geo/query/acc.cgi?acc=GSE52588

GSE106648: PBL, Multiple sclerosis with healthy controls

https://www.ncbi.nlm.nih.gov/geo/query/acc.cgi?acc=GSE106648

GSE87640: WB, and MACS isolated CD4+, CD8+, CD14+ leukocytes; WB data were used in Figure 2, the entire dataset was used in Figures 5, 6, and Supplementary Figures 4, 5. Irritable bowel disease with healthy controls

https://www.ncbi.nlm.nih.gov/geo/query/acc.cgi?acc=GSE87640

GSE72774: WB, Parkinson’s disease with healthy controls

https://www.ncbi.nlm.nih.gov/geo/query/acc.cgi?acc=GSE72774

GSE100825: WB, Werner Syndrome with healthy controls

https://www.ncbi.nlm.nih.gov/geo/query/acc.cgi?acc=GSE100825

### Elastic net regression

To construct the small and medium EN models, we first did a random 80/20 split to get a training and testing set respectively. To create our models we used the ElasticNetCV class from the sklearn.linear model library. After standardizing the array data using the sklearn preprocessing function StandardScaler, we used ElasticNetCV’s built-in cross-validation functionality to find optimal values for the regression parameters alpha and l1 ratio (used in Scikit-learn’s EN regression) for each set of data. We used these parameters to also fit the randomized versions of these clocks. We then tested the predictive ability of these models on the test dataset. After the models were trained and tested, we also tested their performance on out-of-sample datasets. We followed the same data preprocessing procedure on out-of-sample datasets.

### DNAme EN predictor of US population

We built a model that was able to predict the growth of US population, using the same dataset, GSE40279, as well as the same training techniques as for in the standard age predictor, but instead of correlating with known ages of the donors of blood samples, this EN model predicts the number of people living in US at the time of the donors’ birth. Although we’re able to accurately predict the US population from the birth year of a sample based on the sample’s methylation data, there of course is no direct connection between one’s methylation data and the US population from their birth year. In other words, the methylation profile of an individual does not determine the US population at their birth.

### Models 0 and 1

The data were imported from the GEO database using the GEOparse package in Python. For GSE40279, kernel density estimation plots for each GSM were compared to identify potential outliers. None were found and all samples were used. The data were split 80/20 into training and test sets respectively. The training set was scaled using StandardScaler in the Scikit-learn library. ElasticNetCV, with 10-fold cross validation (cv=10), was used to train an elastic net regression model. The l1 ratio was set to 0.5 and the alpha hyperparameter was learned from a set of ten using cross validation (n alphas = 10). To check model accuracy the test data were transformed using the training set StandardScaler fit prior to predictions.

### UMAP

We employed conventional unsupervised Machine Learning algorithm and Uniform Manifold Approximation and Projection (UMAP) method to reduce the dimension of 482421 Cytosines (GSE4027) in each individual and compare the closeness between individuals in terms of Cytosines beta value. Proximity in low-dimensional UMAP space identifies groups of individuals that correspond to their health condition or disease type.

### Retraining an elastic net model after removing non-significant cytosines

The significant Cytosines of the elastic net model were identified by their non-zero weights. A modified dataset was made by removing these significant Cytosines and a new model was trained and tested with this modified dataset. We first trained an Elastic Net model, using the ElasticNetCV function from the “sklearn” python library with a trained alpha value, a L1 value of 0.5, a tolerance of 0.001, and a maximum iteration of 5000, on the GSE40279 dataset used by the DNAm Clock developed by Hannum et al. and found that this model relies on 849 Clock cytosine’s. We then randomly removed non-clock cytosine’s from our dataset and retrained Elastic Net with the same parameters on datasets with 1-10% of non-clock cytosine’s removed in 1% intervals and on datasets with 10-100% of non-clock cytosine’s removed in 10% intervals. These novel datasets are created by independently removing, by selecting random integers with the “random” python library, the desired percent of cytosine’s from the original list of non-clock cytosine’s. These newly trained Elastic Net models are then simply analyzed with the python libraries “Sklearn”, “matplotlib”, and “pandas” to create Figure 3 and Supplementary Figure 3.

### Gene annotations for cytosines, gene functions and families

The gene/genome location of the cytosines and their annotations were taken from the Illumina HumanMethylation450 manifest. The gene functions and families were determined from the GeneCards and Entrez databases (https://www.genecards.org/, https://www.ncbi.nlm.nih.gov/Web/Search/entrezfs.html).

### Noise barometer

In approach one, to identify cytosines which are on average age-invariant but are age-deregulated, we performed regression analysis and selected cytosines with an individual correlation with age of 0.02 < r < 0.05. This yielded 6,282 cytosines out of 480,000. The mean beta values for each of the 6,282 cytosine was calculated and the absolute difference from the mean (ADM) was calculated (Equation 6, 7). Then for each cytosine, age was regressed on the ADMs and Cytosines with r < 0.2 were discarded. This yielded a set of 50 cytosines. The ADMs (surrogate for noise) of the 50 cytosines were summed for the young samples, and a normalizing factor (ζ) was calculated by taking the average of these sums (Equation 8). A final noise score σ for all samples was calculated by summing the ADMs for each sample and then normalizing by ζ (Equation 9). Y axis biological age numbers were calculated through the polynomial fit for the median value of normalized sum for each age in the healthy control dataset. We set the noise score to be Y and find root, or the X values, from the polynomial equation.

In approach two, to identify cytosines which are on average age-invariant, we calculated the Means of beta values of all cytosines of the six independent 450K array datasets, in the healthy cohorts, and selected the cytosines that had nearly same (>0.1% difference) Means in the young (25-28 years) and the old (<67 years) groups. This selection was then cross-checked by having Pearson’s (r) 0.02-0.05 of beta values over age, e.g., excluding the zeroes of experimental errors; this yielded 460 cytosines out of 480,000 that had such near same Mean in all six studies with the 450K DNAme arrays, in healthy subjects. These 460 cytosines where then narrowed down to those in all six datasets, healthy subjects, with the SD of 0.015 of the absolute and relative (set to 1) Means in the young cohort (highly regulated 8 and 5 cytosines) and those with the SD of 0.3 of the absolute and relative (set to 1) Means in the young cohort (least regulated 17 and 13 cytosines).

In approach three: Two filters were used: 1. Unequal variance, e.g., the heteroskedasticity test (White’s test) for each cytosine, finding all with significant (p < 0.05) unequal variance between old and young (there were 372509 of such cytosines); 2. Old (67+ years of age) SD > Young (25-28 years of age) SD by at least 20%; after the first plus second filters, 143448 cytosines remained from the 480k cytosines, e.g., the typical number of probes of the 450K DNA me array. This approach was also compared with summing SD of all 480K cytosines, e.g., the entire 450K DNAme array data. When analyzing the 143448 cytosines in the six combined datasets, some samples of the DNAme array had missing values for a few cytosines in some datasets; to ensure data consistency, all data for such samples was removed from the analyses and the plots. Polynomial curves were fit to the values, using np.polyfit function.

### Direct test of the batch effect

Polynomial curve was fitted to the chronological age v. Median of the 293 summed SDs (the 293 cytosines of the 460 group have at least 20% higher SD in the old than young cohort). Healthy control datasets of arthritis, Multiple Sclerosis and Parkinson’s were used for generating the Median curve and overlaying it with the scatter plot of each individual dataset. The three datasets were chosen because they have the most samples out of all other studied here datasets.

### Availability of data and materials

All data sets used in this study are publicly available on NCBI’s GEO database, found at https://www.ncbi.nlm.nih.gov/geo/.

Equations

**Table:** 

$\begin{array}{l} C(X,y,w,\alpha ,\rho )=\;\frac{1}{2n}{\left\|Xw-y\right\|}_{2}^{2}+\alpha \rho {\left\|w\right\|}_{1}\\ +\frac{\alpha (1-\rho )}{2}{\left\|w\right\|}_{2}^{2} \end{array}$	(1)
$\hat{w}=\underset{w}{min}\;C(X,y,w,\alpha ,\rho )$	(2)
$prediction_{i}=\hat{w}•x_{i,*}$	(3)
$r=\frac{\sum (x_{i}-\bar{x})(y_{i}-\bar{y})}{\sqrt{\sum {\left(x_{i}-\bar{x}\right)}^{2}\sum {\left(y_{i}-\bar{y}\right)}^{2}}}$	(4)
$MedAE(y,\hat{y})=median\left(\left|y_{1}-{\hat{y}}_{1}\right|,\ldots ,\left|y_{n}-{\hat{y}}_{n}\right|\right)$	(5)
$\mu_{j}=\;\frac{1}{N}\sum_{i}\beta_{ij}$	(6)
${\bar{x}}_{ij}=\left|\beta_{ij}-\mu_{j}\right|$	(7)
$\zeta =\frac{1}{m}\sum_{k}\sum_{j}{\bar{x}}_{kj}^{*}$	(8)
$\sigma_{i}=\frac{1}{\zeta }\sum_{j}{\bar{x}}_{ij}$	(9)

## Supplementary Material

Supplementary Figures

Supplementary Data Excel
