# Supplementary material for: Fail-tests of DNA methylation clocks, and development of a noise barometer for measuring epigenetic pressure of aging and disease
Source: Aging (Albany NY). 2023 Sep 12;15(17):8552–75. doi: 10.18632/aging.205046 (PMC10522373; doi:10.18632/aging.205046)
Supplement: Supplementary Figures [file aging-15-205046-s001.pdf]

SUPPLEMENTARY FIGURES

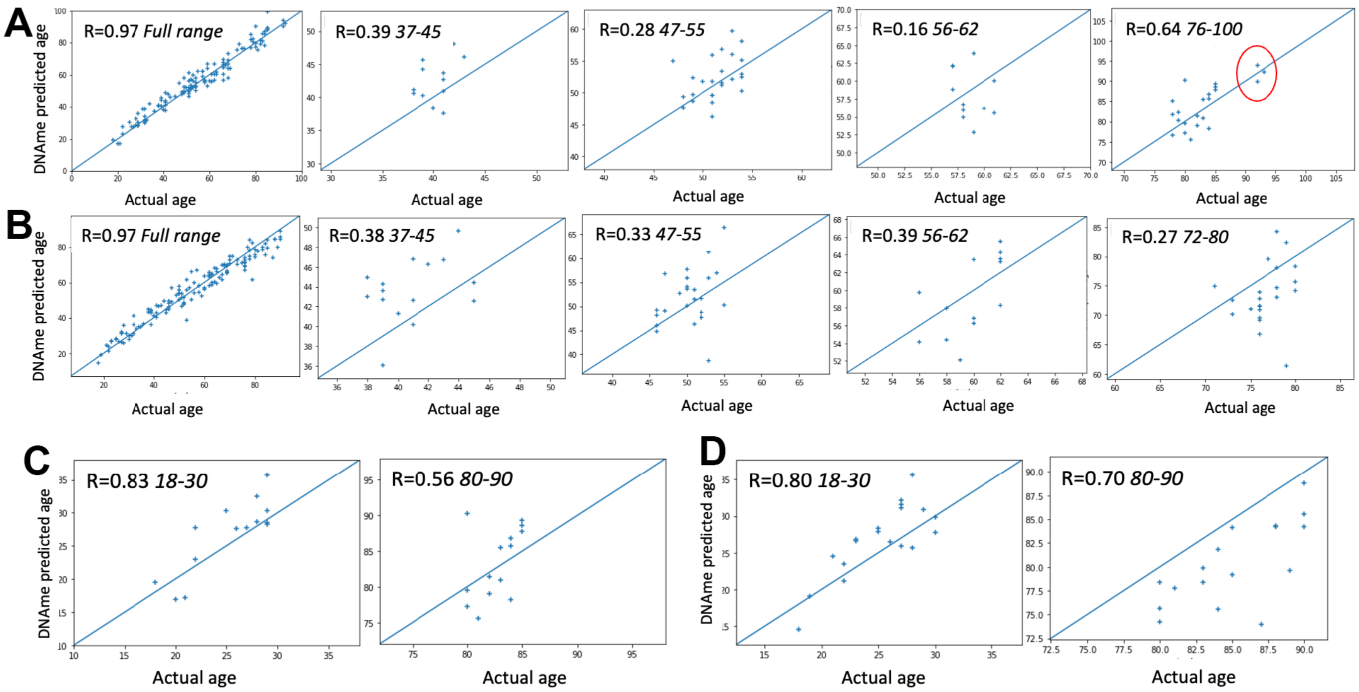

Supplementary Figure 1. The age-region linearity of the EN models. (**A**) A standard DNAme clock was trained on the beta values and sample ages from GSE40279. The data were split 80/20 into training and test sets respectively. The predictions and their correlations (Pearson's r) on the test data over the full chronological range and on specific age ranges (*italicized)* are shown. (**B**) The sample numbers of all age regions were balanced, the DNAme clock was trained and tested. The most linear age regions of 18-30 year of age and 80-90 years of age are shown for the standard (**C**) and the age-even (**D**) models. Interestingly, in the age-even EN model for the very old age region, all samples are under-predicted, Figure 1D. The Pearson's r of 0.64 of the 76-100 yr. range is potentially lower if not for the 80-95ya transition (outlined in red in **A**). These age-ranges were selected to test for the presence of DNAme plateaus, which might be less visible at the low magnification of the total age range.

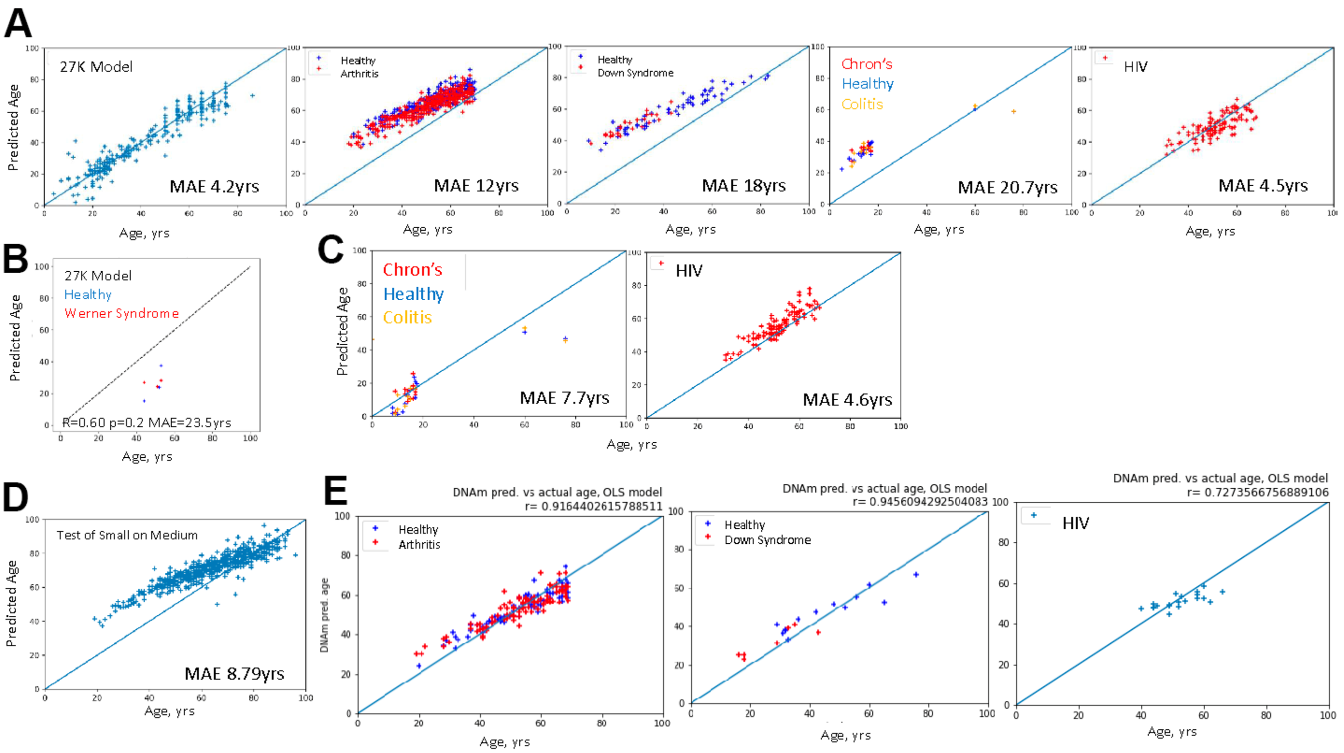

Supplementary Figure 2. Testability of 27K and 450K EN models on additional disease/health datasets, and a demonstration that simple OLS on clocks cytosines is accurate on out-of-sample datasets. (**A**, **B**) Tests of the 27K models on out-of-sample datasets with disease cases, as indicated. (**C**) Tests of the same 450K model as in Figure 1 on out-of-sample datasets with disease cases, as indicated. The ages for healthy subjects (blue) and patients (red) overlap. (**D**) Test of 27K model on 450K dataset yields age-acceleration for most of the samples. (**E**) Simple OLS on clock cytosines is functional for age predictions and overlaps health (blue dots) and disease (red dots). GSEs are provided in Methods.

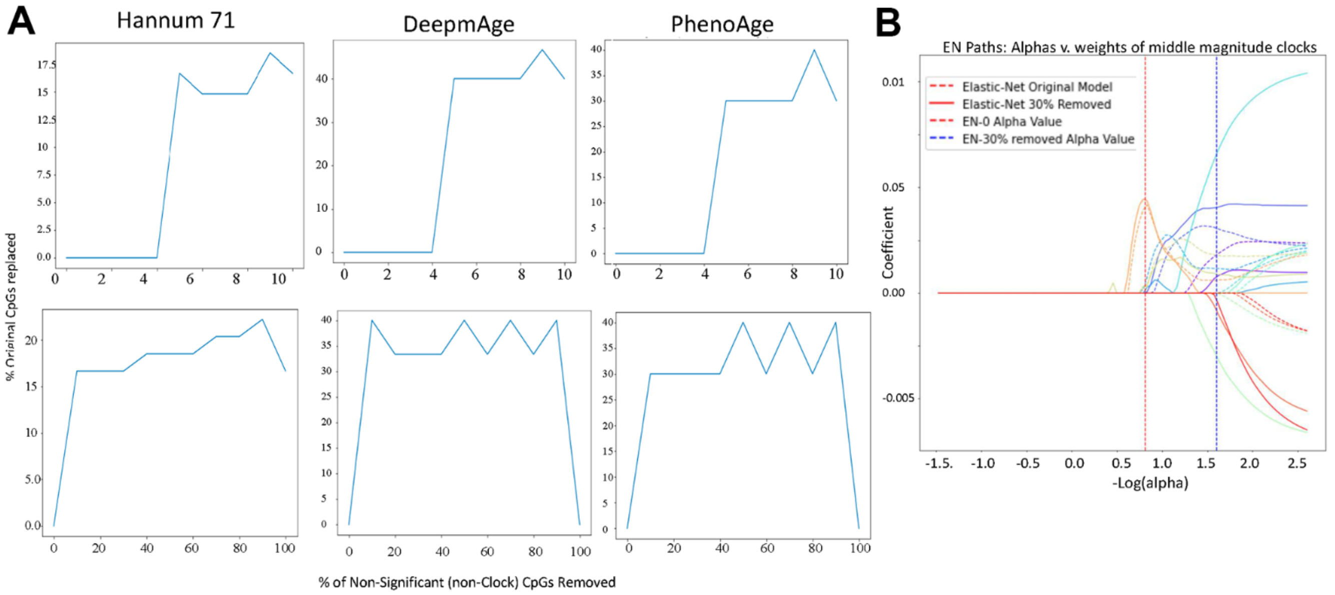

Supplementary Figure 3. Next gen clocks are not resilient to the removal of non-clock cytosines, and cytosine selection likely reflects changes in the data landscape. (**A**) Standard EN clock was trained on the Hannum dataset (GSE40279), after which non-clock cytosines were independently stepwise removed and the alterations to the putatively relevant features of Hannum 71, DeepmAge and PhenoAge clocks were analyzed. Percent clock cytosines removed is shown at the removal of 1-10% and 10-100% of non-clock cytosines. (**B**) A demonstration of the effect of removing the non-significant cytosines has on the Elastic-Net energy landscape. Coefficients assigned to 10 middle value clock cytosines as a function of the alpha hyperparameter significantly differ in both the original EN model and a model with 30% of non-significant cytosines removed across alpha. There are significant differences between the coefficients the models assign, and the models eventually settle to and between these alphas.

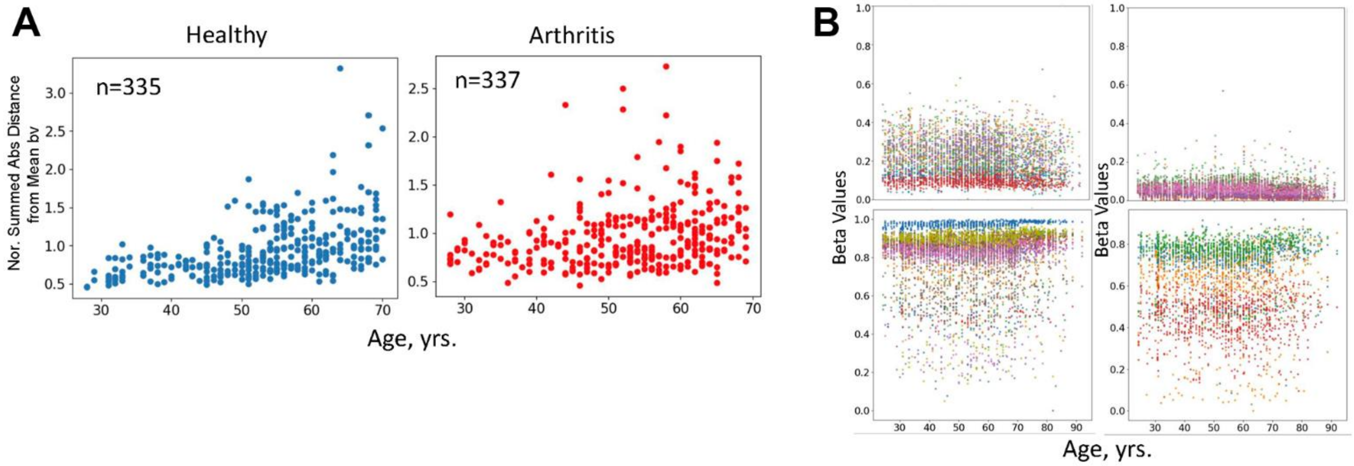

Supplementary Figure 4. Raw data on the 50-cytosine noise barometer and on Low-mid, high methylated and unmethylated cytosines from the 17-cytosine group. (**A**) The normalized by young age, summed absolute distance from Mean of beta values v. chronological ages are shown as the scatter plots, each dot is an individual. Blue – healthy controls, Red – patients with arthritis. Polynomial fit of the Median is in Figure 4B. (**B**) Shown are the beta values over age scatter plots of the for the additional to Figure 4 cytosines that nave near same Mean of beta values in young and old cohorts and are the least regulated in the young cohort (SD>0.3 of Mean). Each dot is a sample, each cytosine is color coded.

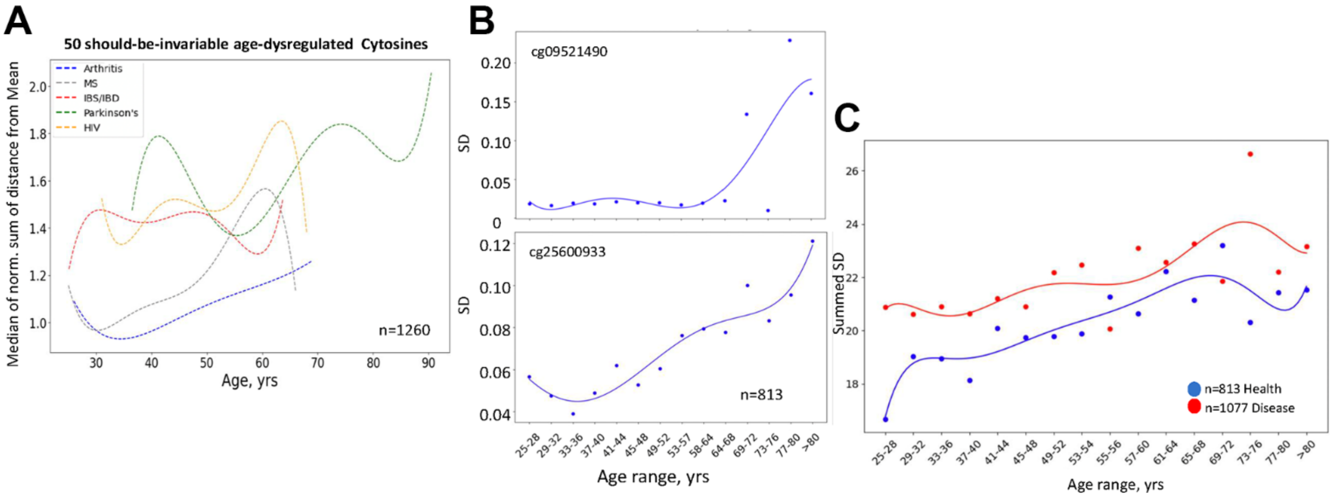

Supplementary Figure 5. Biological noise curves for individual diseases, individual cytosines, and the combined health v. disease progression of biological aging with HIV dataset included. (**A**) The 50-cytosine based measurement of biological noise is shown for the indicated diseases. The shapes of the cures are relevant in this panel. (**B**) Individual highly regulated in young cytosines have different curves of biological aging. (**C**) The shown Health (blue) versus Disease (red) polynomial fit curves of biological aging are based on the 460 cytosines and include HIV dataset, which does not have its own healthy control. In (**B**, **C**), each dot is an age-interval.
